# Lithium-magnetic resonance imaging in bipolar disorder: non-invasive, direct, *in vivo* imaging of a drug in its target organ

**DOI:** 10.1093/psyrad/kkaf017

**Published:** 2025-06-09

**Authors:** Peter E Thelwall, David A Cousins

**Affiliations:** Translational and Clinical Research Institute, Newcastle University, Newcastle upon Tyne, NE2 4HH, UK; Newcastle Magnetic Resonance Centre, Newcastle University, Newcastle upon Tyne, NE4 5PL, UK; Translational and Clinical Research Institute, Newcastle University, Newcastle upon Tyne, NE2 4HH, UK; Newcastle Magnetic Resonance Centre, Newcastle University, Newcastle upon Tyne, NE4 5PL, UK; Cumbria, Northumberland, Tyne and Wear NHS Foundation Trust, Newcastle upon Tyne, NE3 3XT, UK

Lithium is the best-evidenced long-term medication for bipolar disorder, reducing episodes of illness, hospital admissions, all-cause mortality and suicidal behaviour (Cipriani *et al*., 2013; Post & Rybakowski, 2024). A good response is typically observed in a third of patients, with a poor response in a similar proportion (Garnham *et al*., 2007; Hou *et al*., 2016). Given the burden of serum monitoring and lithium's adverse effects on renal, thyroid and parathyroid function, the ability to predict response would improve outcomes and lessen the risks of unnecessary exposure. Clinical predictors of response include the polarity sequence, fully remitting course, number of hospitalizations, age of onset and family history of response (Gitlin & Bauer, 2024), with prospective and in-depth clinical characterization central to research initiatives and implementation in practice (Duffy & Grof, 2024). Relying solely on predictors dependent on illness course may delay the initiation of lithium, potentially detracting from the benefits of early use (Kessing *et al*., 2014). A biomarker of response, measurable before or not long after treatment initiation, is sorely needed and has long been sought (Grof *et al*., 2009).

In this issue of *Psychoradiology*, Boere and colleagues present the protocol for their observational, longitudinal study of patients with bipolar disorder in routine care who have recently started lithium and attained therapeutic serum concentrations (Boere *et al*., 2025). Participants will undergo magnetic resonance imaging (MRI) scans on a high-field (7T) scanner delivering conventional anatomical imaging and lithium chemical shift imaging (^7^Li-CSI) to yield brain lithium distribution. The imaging data, obtained within a month of dose stabilization, will be analysed with respect to response to treatment as determined using the Alda scale (Grof *et al*., 2002) applied after 1 year of treatment.

Just as conventional diagnostic MRI exploits the magnetic properties of hydrogen nuclei in water to image anatomical structure, ^7^Li-MR detects MR-visible lithium nuclei and can report on their concentration in brain tissue. Implementation is challenging, because the concentration of lithium ions in the brain at therapeutic levels is approximately five orders of magnitude lower than the concentration of hydrogen nuclei in tissue water. Further, the MR signal from ^7^Li nuclei is about a quarter of the magnitude of an equivalent number of ^1^H nuclei. In short, ^7^Li-MRI has a sensitivity problem which manifests as low-resolution images with long scan times. Nevertheless, human brain ^7^Li-MR scan methodology has evolved during the four decades since the first report of non-localized detection from *in vivo* human brain (Renshaw & Wicklund, 1988). Volume-localized spectroscopy, spectroscopic imaging, gradient echo imaging and balanced stead-state free precession MRI methods have brought progressive improvements in spatial resolution and/or scan duration (Gonzalez *et al*., 1993; Girard *et al*., 2001; Soares *et al*., 2001; Boada *et al*., 2010; Machado-Vieira *et al*., 2016; Smith *et al*., 2018). The evolution of MR hardware, bringing higher scanner magnetic field strengths, improved RF coil (sensor) performance and scanner architecture improvements, have delivered substantial advances in ^7^Li-MRI scan performance, such that 3D maps of brain lithium content can be acquired in scan durations acceptable for clinical research. There is ongoing scope for further ^7^Li-MRI methodological refinement, whilst analysis strategies have also evolved, permitting the colocalization of brain lithium concentration and its tissue-level effects on white matter integrity (Necus *et al*., 2019). The potential of lithium imaging is supported by studies demonstrating differences in the relationship between brain and serum lithium concentrations in responders and non-responders (Machado-Vieira *et al*., 2016).

Boere *et al*. join a growing field of ^7^Li-MRI centres and studies that seek to understand the relationship between brain lithium and the efficacy of treatment. The most directly comparable study is the EU Horizon 2020-funded R-LiNK consortium (https://rlink.eu.com), which has now concluded data collection. A primary objective of the R-LiNK study was to optimize the early prediction of lithium response using a set of multimodal biomarkers, including blood -omics, anatomical and diffusion-weighted MRI, brain ^1^H-MR spectroscopy and ^7^Li-MRI (measured using a balanced steady-state free precession scan) (Scott *et al*., 2019). Lithium was initiated and monitored as part of routine care, with biomarker measurement prior to initiation and after 3 months of treatment. Monthly clinical research assessments were performed over a 2-year follow-up period and used in rating response with the Alda scale at the close of data collection. Within the broader network of R-LiNK centres, ^7^Li-MRI was established and harmonized across six European sites, five of which used 3T MRI scanners (across two vendor types) and one which used 7T MRI.

Boere *et al*.'s study will generate the largest longitudinal lithium imaging cohort at high scanner field strength to date, drawing on a patient population within a single healthcare system. Should brain lithium distribution prove sufficient as a predictor of response, these methods ultimately have potential for translation to clinical practice, supported by scanner manufacturers increasingly incorporating multinuclear MR into the routine clinical capabilities of their products. The follow-up period of 1 year may make it more difficult to be certain of relapse prevention in subjects with a pattern of infrequent relapses but may substantially improve our understanding of early response prediction, which may of course be sustained. Importantly, the predictive potential of a biomarker is intimately related to the evaluation of response in development and validation studies. Longitudinal, prospective assessment with close attention to the impact of lithium on clinical course, degree of adherence and the adequacy of serum lithium concentrations will be key determinants of the success of such endeavours.

It is worth reflecting that despite being our oldest drug, lithium remains at the vanguard of advances in psychopharmacology. The delineation of excellent lithium responders provides the ideal framework for the development of patient stratification and personalized interventions in bipolar disorder, a common and often devastating major mental illness. Brain lithium imaging represents the direct detection and quantification of the distribution of a drug in its target organ – a rare feat in medicine, let alone psychiatry. Beyond the promise of response prediction, we anticipate advances in the understanding of lithium's actions *in vivo*, insights into tolerability and toxicity, tissue-level explorations of dosing regimens and therapeutic concentrations, as well as the application to a broader range of conditions such as neurodegenerative disorders. These approaches will foster personalized management, better outcomes and greater patient engagement in the choice to consider lithium as a long-term treatment.

## Notes

Commentary on “The Bipolar Lithium Imaging Scan Study (BLISS): protocol for a 7T lithium-7 magnetic resonance study in bipolar disorder”—Boere et al.
